# CD8 T-Cells from Most HIV-Infected Patients Lack Ex Vivo HIV-Suppressive Capacity during Acute and Early Infection

**DOI:** 10.1371/journal.pone.0059767

**Published:** 2013-03-29

**Authors:** Camille Lécuroux, Isabelle Girault, Antoine Chéret, Pierre Versmisse, Georges Nembot, Laurence Meyer, Christine Rouzioux, Gianfranco Pancino, Alain Venet, Asier Sáez-Cirión, ANRS 147 OPTIPRIM clinical trial

**Affiliations:** 1 INSERM, U1012, Le Kremlin Bicêtre, France; 2 Hôpital Gustave Dron, Service de Maladies Infectieuses et du voyageur, Tourcoing, France; 3 EA 3620, Université Paris-Descartes, Sorbonne Paris Cité, Paris, France; 4 Institut Pasteur, Unité de Régulation des Infections Rétrovirales, Paris, France; 5 INSERM U1018, Université Paris-Sud 11, Le Kremlin Bicêtre, France; 6 AP-HP, Hôpital de Bicêtre, Département d’épidémiologie, Le Kremlin Bicêtre, France; 7 AP-HP, CHU Necker-Enfants Malades, Laboratoire de Virologie, Paris, France; Massachusetts General Hospital, United States of America

## Abstract

The strong CD8+ T-cell-mediated HIV-1-suppressive capacity found in a minority of HIV-infected patients in chronic infection is associated with spontaneous control of viremia. However, it is still unclear whether such capacities were also present earlier in the CD8+ T cells from non controller patients and then lost as a consequence of uncontrolled viral replication. We studied 50 patients with primary HIV-1-infection to determine whether strong CD8+ T-cell-mediated HIV suppression is more often observed at that time. Despite high frequencies of polyfunctional HIV-specific CD8+ T-cells and a strong CD4+ T-helper response, CD8+ T-cells from 48 patients lacked strong HIV-suppressive capacities ex vivo. This indicates that the superior HIV-suppressive capacity of CD8+ T-cells from HIV controllers is not a general characteristic of the HIV-specific CD8+ T cell response in primary HIV infection.

## Introduction

During the acute phase of HIV-1 infection the virus spreads rapidly through the body and plasma viremia rises exponentially to high levels. Viremia starts to decline gradually three weeks after infection, reaching a stable level a few months later. This “steady state” viremia varies from one individual to another and is predictive of the rate of disease progression. The fall in plasma HIV viremia during the acute infection coincides with the emergence of HIV-specific CD8+ T-cells [1], which exert selection pressure on the virus, forcing it to evolve to elude recognition [2]. In vivo depletion of CD8+ cells in macaques during primary SIV infection abrogates their ability to control primary viremia [3]. These findings suggest that the CD8+ T response is involved in the initial control of viral replication during primary HIV-1 infection (PHI).

HIV-specific immune responses deteriorate as the infection becomes chronic. In particular, HIV-specific CD4+ helper T-cells become dysfunctional [4] and HIV-specific CD8+ T-cells also gradually lose several functions (including their proliferative capacity, cytotoxic potential, and capacity to produce IL-2 and other cytokines [5]), and become senescent [6]. In many rare “HIV controllers” (HIC), in whom viremia remains undetectable without antiretroviral therapy, highly functional HIV-specific CD8+ T-cells are maintained. These cells are able to produce several cytokines and to proliferate upon antigen stimulation [7], [8], even more than ten years after initial infection. CD8+ T-cells from these HIC have an impressive capacity to suppress HIV infection of autologous CD4+ T-cells [9]. This capacity is related to a high frequency of HIV-specific CD8+ T-cells, including those targeting epitopes in Gag [10], and also to their high lytic granule content [11], [12]. HIC are a heterogenous population and some of them have very weak HIV-specific T cell responses [13], [14], [15], pointing to the existence of additional mechanisms contributing to control infection. Nevertheless, it is believed that this efficient CD8+ T-cell response plays an important role in the spontaneous virus control in many HIC.

It is unclear whether the superiority of HIC CD8+ T-cells to suppress the virus is due to intrinsic characteristics or simply reflects the loss of functional capacity due to persistent viral replication in non-controllers. To address this question, we studied 50 individuals recently infected with HIV-1, focusing on the frequency of HIV-specific T-cells, their potential to produce several cytokines, and the capacity of CD8+ T-cells to control infection of CD4+ T-cells ex vivo.

## Materials and Methods

### Patients

**Figure 1:**
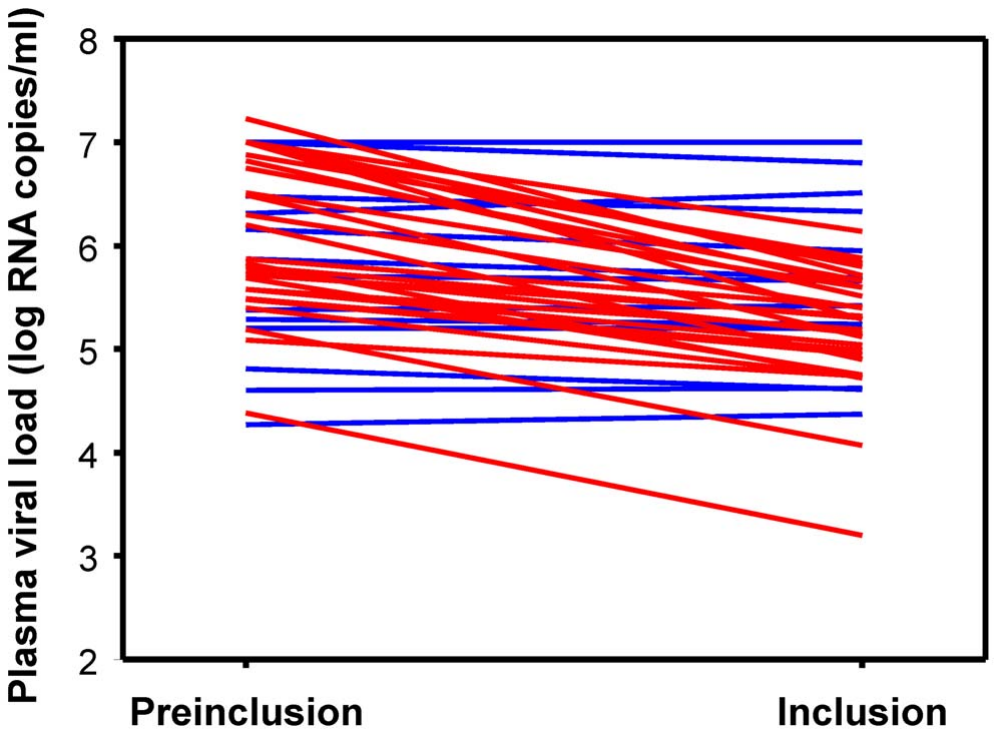
Time course of plasma viral load in PHI patients between the pre-inclusion visit (∼d-7) and inclusion of the study (d0). Values for patients who experienced a decline of at least 0.3 log in their viral load (arbitrary threshold) during this period are shown in red, and those from patients whose viral load remained stable are in blue.

Fifty participants in the ANRS 147 OPTIPRIM clinical trial were included in this study (Table 1). OPTIPRIM is a multicentre, phase 3 randomized trial designed to examine the impact, after 24 months, of maximized versus conventional combination antiretroviral therapy (cART) on HIV reservoirs in patients with acute or early primary HIV-1 infection (ClinicalTrials.gov ID: NCT01033760). The 50 study participants were recruited between 2010 and 2011, within ten weeks of diagnosis of symptomatic PHI. Acute infection was defined by a negative or weakly positive HIV-1 Elisa plus a negative or incomplete (1 antibody) HIV-1 Western blot, and HIV-1 RNA and/or p24 antigen positivity. Early infection was defined by a positive HIV-1 Elisa plus an incomplete Western blot (≥2 and <5 antibodies, with the presence of anti-p24 and anti-gp160, -gp120 or -gp41 reactivity) and HIV-1 RNA positivity. The date of infection was estimated as the day of symptom onset minus 15 days, and the interval between infection and inclusion in the study was 35 days [31–43] (median and interquartile range (IQR)). Most of the patients were men (n = 47). Age at inclusion was 38 years [29–47]. CD4+ T-cell counts and plasma viral loads at inclusion were 466 [362–652] cells/µl and 5.42 [4.99–5.88] log HIV-1 RNA copies/ml. An additional viral load determination, obtained a median of 7 [6]–[10] days before inclusion in the study, was available for 49 patients with PHI. These viral loads were, in general, higher (5.87 [5.4–7.00] log HIV-1 RNA copies/ml, P<0.001) than at inclusion in the study. Viral load in these patients declined by at least 0.3 log in 27 patients (median -0.94 log HIV-1 RNA copies/ml [0.54–1.19]) during this short period (Figure 1). This showed that, at the time of the study, these patients were in the descending phase of viremia that follows peak viral load during PHI. All samples were obtained before the patients initiated treatment.

**Table 1:** Characteristics of groups of patients included in the study.

Characteristics (median [IQR])	PHI^1^ patients from ANRS OPTIPRIM trial	HIC^2^ from ANRS CO18 cohort
Number of patients	50	46
Days since infection	35 [31–43]	>1825
Fiebig Stage	IV [III–V]	NA^3^
**HLA**		
% of B*27 patients	2	9
% of B*57 patients	2	35
Age	38 [28–47]	46 [40–52]
Log HIV RNA VL (copies/ml)	5.42 [4.99–5.88]	<1.6[<1.3–1.7]
CD4 T cells (cells/µl)	466 [362–652]	841 [759–1114]
CD8 T cells (cells/µl)	1124 [715–1654]	840 [640–1018]

1 PHI, Primary HIV Infection;

2 HIC: HIV controllers;

3 NA: not applicable.

We compared CD4 and CD8 T-cell responses from patients with PHI to those of 46 HIC from the ANRS CO18 HIV controller cohort (Table 1). HIC were defined as patients who had been infected for more than 5 years and whose last five consecutive plasma viral loads were below 400 HIV-1 RNA copies/ml of plasma. Respectively 16 and 4 HIC carried one protective allele (HLA-B*57 or B*27), and three HIC carried both alleles.

### Ethics Statement

All the subjects gave their written informed consent to participate in the study. This study is developed in the context of the ANRS 147 OPTIPRIM clinical trial (ClinicalTrials.gov ID: NCT01033760) and is sponsored by the French National Agency for research on AIDS and viral hepatitis (ANRS) and was approved by the Sud Méditerranée I ethics review committee and the French Health Products Safety Agency (AFSSAPS).

### Cell Sampling and HLA Typing

Peripheral blood mononuclear cells (PBMC) were isolated from EDTA-anticoagulated blood by Ficoll density gradient centrifugation, and were used fresh (phenotypes, HIV-suppression assay) or stored in liquid nitrogen (intracellular cytokine production). Human leukocyte antigen typing used the complement-dependent microlymphocytotoxic technique (Ingen).

### Detection of Intracellular Cytokine Production

Frozen PBMC were thawed in RPMI medium containing 10% fetal calf serum and antibiotics and were stimulated for 15 hours in medium containing the relevant Env, Gag, Pol or Nef optimal peptide pools (1 µg/mL) according to the subjects’ HLA type, or P24 (1 µg/mL), in the presence of brefeldin A (10 µg/mL) (Sigma-Aldrich). After incubation, samples were stained for viability with the LIVE/DEAD® Fixable Violet Dead Cell Stain kit (Invitrogen) and then with the following antibodies to quantify cytokine production in CD8+ and CD4+ T-cells: anti-IL-2-phycoerythrin (PE), -MIP-1β-peridin chlorophyll protein-cyanin 5.5 (PerCP-Cy5.5), -CD3-PE-cyanin 7 (PE-Cy7), -IFN-γ-allophycocyanin (APC) and -CD8-APC-H7 (Becton Dickinson). A negative control (medium) and a positive control (staphylococcal enterotoxin B, SEB) were included in each experiment. Samples were acquired on a BD LSRFortessa™ flow cytometer (Becton Dickinson) and analyzed with DIVA software (Becton Dickinson).

### Measurement of CD8 T-cell-mediated HIV Suppression ex vivo

The method used to assess the capacity of CD8+ T-cells to suppress HIV-1 infection of autologous CD4+ T-cells ex vivo is described in detail in [16]. Briefly, CD4 and CD8+ T cells were isolated from freshly purified PBMC by, respectively, positive and negative magnetic bead-sorting (Stemcell Technologies). CD4+ cells were activated with phytohemagglutinin (1 µg/ml) and IL-2 (100 UI/ml) for three days. CD4+ T cells were then infected in vitro with HIV-1 BaL using a spinoculation protocol [17] and cultured alone or co-culture with autologous CD8+ T cells at a 1∶1 ratio. Non superinfected CD4+ T cells were also cultured in parallel to assess replication of autologous virus. Viral replication was measured by p24 production in culture supernatants by ELISA (Zeptometrix).The index of in vitro superinfection for each experiment was determined by comparing the peak level of p24 in culture supernatants of PHA-activated CD4+ T cells infected in vitro with HIV-1 BaL to that of culture supernatants of PHA-activated CD4+ T cells not exposed to HIV-1 BaL. Capacity of CD8+ T cells to suppress HIV infection was calculated as the Log drop in p24 production when superinfected CD4+ T cells were cultured in presence of CD8+ T cells. Experiments were conducted in triplicate with cells from each patient.

### Statistical Analyses

The Mann-Whitney rank sum test was used to compare variables between groups. Correlations were evaluated by using simple linear regression analysis and Spearman’s rank correlation test. All values given in the text are medians and [IQR]. SigmaStat 3.5 software (Systat Software Inc.-SSI, CA) was used for all analyses.

## Results

### Robust HIV-specific CD4+ and CD8+ T-cell Responses, Including IL-2 Production, during PHI

**Figure 2:**
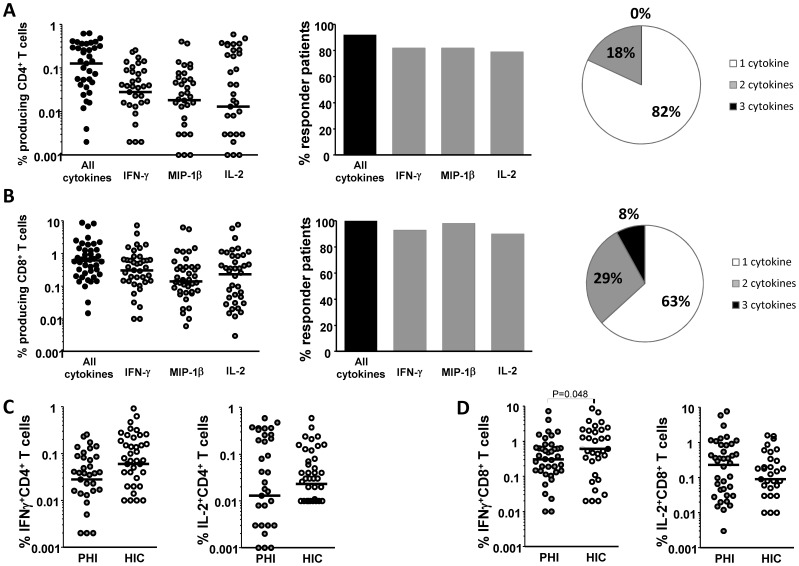
Similar HIV-specific T cell responses in PHI-patients and HIC. **A.** PHI patients: frequency of CD4+ T-cells producing at least one cytokine (IFN-γ, MIP-1β or IL-2) in response to HIV-p24 stimulation, as determined by ICS (left); percentage of patients with a positive CD4+ T-cell response (centre); and frequency of HIV-specific CD4+ T-cells producing one, two or three cytokines (right) **B.** same experiments with CD8+ T-cells challenged with MHC-matched optimal HIV-1 peptides. **C.** Comparative frequency of IFNγ- (left) and IL2 (right)-producing CD4+ T-cells in PHI patients and HIC. D. *Idem* for CD8+ T-cells. Each symbol represents one individual. Medians are shown as horizontal lines.

We detected HIV-specific CD4+ T-cells responding to p24 in 36 (92%) of 39 individuals tested; a median of 0.13% [IQR: 0.03%-0.31%] of CD4+ T-cells produced at least one cytokine (IFNγ MIP-1β and/or IL-2) (Figure 2A and Figure S1A). The most frequent responses were observed for IFNγ and MIP-1β production, which was observed in 82% of patients (Figure 2A, center). Interestingly, IL-2-producing CD4+ T-cells were detected in 31 of the 39 patients tested and represented the largest fraction of responding CD4+ T-cells in 13 patients, with a median of 0.01% [0.001–0.21] of CD4+ T-cells (Figure 2A, left). Eighteen per cent of all responding cells were able to produce two cytokines (IL-2+ MIP-1β in 10% of cells and IL-2+ IFNγ in 6% of cells) (Figure 2A, right). None of these patient’s CD4+ T-cells were able to produce the 3 cytokines simultaneously.

As expected, high frequencies of HIV-specific CD8+ T-cells were also detected in all 42 patients studied (median frequency 0.61% [0.24%–1.29%]), along with significant IL-2 responses (90% of patients, median frequency 0.23% [0.03%–0.77%]) (Figure 2B and Figure S1B). Among responding cells, 37% were able to produce at least two cytokines (Figure 2B, right). The highest frequencies concerned nef peptides, followed by gag, pol and env (data not shown).

We then compared these responses to those observed in HIC. Previous analyses of the ANRS HIC cohort have shown that many HIC have strong HIV-specific CD8 T-cell responses, comparable to those observed in viremic patients, as well as preserved T helper responses able to produce IL-2 [9], [18]. Frequencies of HIV-specific CD4+ T-cells producing IFNγ or IL-2 were similar in PHI patients and HIC (0.03% [0.004%–0.08%] vs 0.06% [0.01%–0.16%] IFNγ-producing CD4+ T-cells, respectively; 0.01% [0.001%–0.21%] vs 0.02% [0.01%–0.07%] IL-2-producing CD4+ T-cells, respectively) (P>0.05 for both comparisons) (Figure 2C). The frequency of IFNγ-producing CD8+ T-cells was slightly higher in HIC (Fig 1D) (0.61% [0.27%–2.02%] vs 0.30% [0.12%–0.64%] in PHI patients, P = 0.045). However, many patients with PHI had frequencies similar to those observed in HIC (Figure 2D). Moreover, no differences were found between PHI patients and HIC in terms of HIV-specific CD8+ T-cells able to produce IL-2 (0.23% [0.03%–0.77%] vs 0.09% [0.03%–0.3%], respectively; P = 0.34).

Thus, most patients with PHI possessed both multifunctional HIV-specific CD8+ T-cells and helper CD4+ T-cells able to produce IL-2, with no evidence of exhaustion.

### CD8+ T-cells from most Patients with PHI lack Strong Capacity to Suppress HIV Replication

The capacity of CD8+ T-cells to eliminate infected CD4+ T-cells is one of the best correlates of viral control in vivo [12], [16], [19], [20]. We thus explored the capacity of purified CD8+ T-cells from 48 patients from the ANRS 147 OPTIPRIM study to suppress HIV-1 infection of autologous CD4+ T-cells ex vivo.

**Figure 3:**
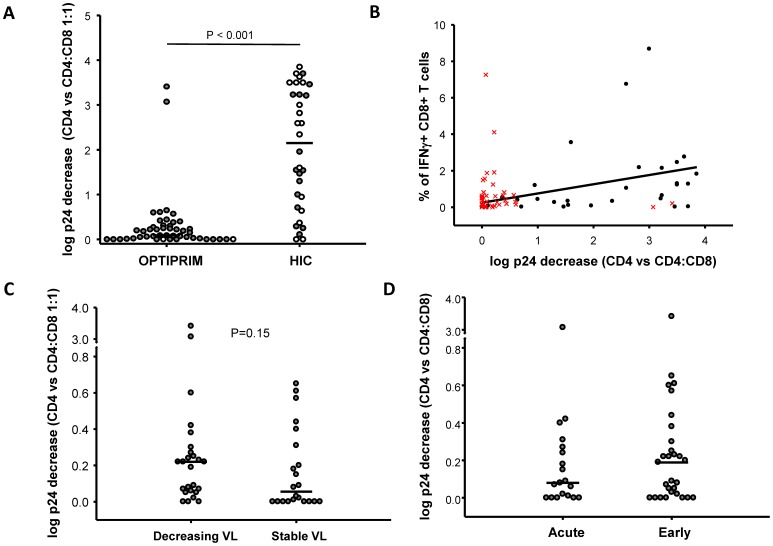
Modest capacity of CD8+ T cells from PHI-patients to suppress infection. **A.** Capacity of CD8+ T-cells from PHI patients and HIC to suppress HIV infection of autologous CD4+ T-cells ex vivo, measured as the log fall in p24 production in culture supernatants when CD4+ T-cells superinfected in vitro with HIV-1 Bal were co-cultured with unstimulated autologous CD8+ T-cells at a ratio of 1∶1, by comparison with p24 production by superinfected CD4+ T-cells cultured alone. Each symbol represents one patient. Open circles represent patients carrying at least one HLA-B*27 or B*57 allele. **B.** Correlation between CD8+ T-cell-mediated HIV suppression and the frequency of IFNγ-producing CD8+ T-cells between HIC (black symbols) and PHI patients (red symbols). Linear regression is shown for HIC. **C.** Capacity of CD8+ T-cells from patients with acute or early PHI to suppress HIV infection ex vivo. **D.** Comparison of the capacity of CD8+ T-cells from PHI patients with decreasing and stable viral loads to suppress HIV infection of autologous CD4+ T-cells.

In general, cells from patients with PHI had poor HIV-suppressive capacity (0.09 [0.01–0.28] log p24 decrease CD4 vs CD8:CD4 1:1 E:T ratio), and were far less potent in this respect than cells from HIC (2.15 [0.83–3.35], n = 32 HIC, p<0.001) (Figure 3A). The difference was still highly significant when HIC carrying the protective allele B*57 or B*27 were excluded from the analysis (1.54 [0.78–3.23], n = 15 B*27^neg^B*57^neg^ HIC, p<0.001). We have previously shown that the capacity of CD8+ T-cells from HIC to suppress HIV infection of autologous CD4+ T-cells correlates with the frequency of IFNγ-producing HIV-specific CD8 T-cells [10]. Accordingly, a correlation between HIV suppressive capacity and IFNγ production by HIV-specific CD8+ T-cells was observed in the HIC analysed here (Spearman 0.448, p = 0.015), while no such correlation was observed in patients with PHI (Spearman 0.003, p = 0.986) (Figure 3B).

Due to their high viral loads, PHA-activated CD4+ T cells from some patients in PHI produced higher levels of p24 than cells from HIC, which may constitute a confounding factor when assessing changes in the levels of superinfection in vitro with HIV-1 BaL. However, the capacity to suppress infection of CD8+ T cells from PHI patients for whom viral replication in CD4+ T cells after HIV-1 BaL superinfection in vitro was at least three times the one in non superinfected CD4+ T cells (superinfection index >3) (n = 29) (0.2 [0.05–0.42] log p24 decrease CD4 vs CD4:CD8 1:1) was still much weaker than the responses observed in HIC (p<0.001) (not shown). These results did not change when a superinfection index above 10 was used to make the analyses. CD8+ T cells from HIC had higher viability than cells from PHI patients at the time of co-culture with CD4+ T cells (85 [77–92], n = 32 vs 77 [66–83], n = 42, p = 0.002, % of viable cells from HIC and PHI patients respectively). Nevertheless, the capacity of CD8+ T cells to suppress HIV-1 infection did not change when we paired cell viability from PHI patients to those from HIC (0.08 [0.0–0.23] log p24 decrease for experiments with cells from PHI patients with 83 [80–87] % of viability, n = 18).

In general, CD8+ T cells from patients in PHI were not totally devoid of capacity to suppress HIV infection, and this tended to be slightly higher in patients whose viral load was declining at the time of the study than in those whose viral load remained stable (Figure 3C). CD8+ T-cell-mediated HIV-suppressive capacity during PHI did not correlate with plasma viral load at sampling (Spearman −0.16, p = 0.28, not shown). No differences were observed between patients in the acute and early stages of PHI (Figure 3D), and no correlation was found between CD8+ T-cell suppressive capacity and the estimated time between infection and inclusion in the study (Spearman 0.09, p = 0.56, not shown).

CD8+ T-cells from two patients with PHI stood out for their strong capacity to suppress HIV-1 infection of autologous CD4+ T-cells, at levels similar to those found in HIC (Figure 3A). However, viral load in these two patients was not particularly low when compared to the other PHI patients, and neither patient’s HIV-specific T-cells exhibited a particularly high frequency or strong capacity for cytokine production. Neither patient bore the B*27 or B*57 allele. Two other PHI patients bore one of these alleles, but neither of them had a strong CD8+ T-cell-mediated capacity to suppress HIV-1 ex vivo (Figure 3A).

## Discussion

Due to their outstanding capacity to spontaneously control HIV infection, HIC are the focus of intense research. Although many HIC lack such responses during chronic infection, it is thought that control of infection in these individuals was achieved, at least in part, thanks to a highly efficient HIV-specific CD8+ T cell response. This response is often considered as a model in the development of T-cell based HIV-vaccines. In particular, the final objective of HIV-specific CD8+ T cells is to eliminate HIV-infected CD4+ T cells, and we and others have found that the CD8+ T cells with the highest capacity to do so are found among HIC. However, the strong potential of CD8+ T cells from these HIC to eliminate infected cells coincide with the preservation of other properties, such as secretion of multiple cytokines (in particular IL-2), which are lost in chronic infection in the cells of non controllers due to uncontrolled-viremia-driven exhaustion. Because no information was available so far about the capacity of CD8+ T cells from HIV infected patients to suppress HIV infection during PHI, it was possible that the strong capacities found in HIC were just another reflect of a preserved CD8+ T cell response in HIC.

We analyzed the CD8+ T cell response from 50 patients in PHI. Because it is extremely difficult to identify HIC during PHI due to the rarity of this phenotype, we compared these responses to those observed in a group of HIC during chronic infection. Despite comparable frequencies of HIV-specific CD8+ T-cells and CD4+ T helper cells with a preserved capacity to produce IL-2, CD8+ T-cells from the overwhelming majority of patients with primary HIV-1 infection, including those studied during the phase of active viral control, do not exhibit a strong capacity to suppress HIV infection. Actually, strong capacities to suppress HIV infection were only observed in two of the patients included in the study during PHI. Because all these patients immediately started therapy following inclusion, it is unknown whether these two individuals might have been able to spontaneously control infection.

In a recent work, Yang and collaborators have reported that measuring early in infection the capacity of CD8+ T cell to suppress HIV may predict the rate of loss of CD4+ T cells [21]. Although the objective of our work is different of that of Yang et al, and some differences also exist in the methodology (e.g. time of sampling, CD8:CD4 T cell ratio, patients analyzed), our results provide some support to their observations. Although much weaker than observed in HIC, CD8+ T cells from patients in PHI had some capacity to suppress HIV infection. Interestingly, this capacity tended to be higher in those patients who were experiencing an active decline in their viral loads at the time of sampling. Because of treatment initiation we could not further assess the impact of this capacity in the evolution of infection in this group of patients.

In summary, our results suggest that most HIV infected individuals are not able to develop during PHI CD8+ T cell responses with the superior capacity to suppress HIV infection that is later found in many HIC. We may speculate that this capacity of HIC cells may be established early through the selection/development of cells with particular intrinsic characteristics, such as stronger avidity or more rapid degranulation [12], [22].

## Supporting Information

Figure S1 **Representative example of ICS experiments showing production of IL-2 and IFNγ by CD4+ T cells (A) or CD8+ T cells (B) from a patient in PHI, in the absence of stimulation or in response to HIV antigens or to SEB superantigen.**
(PDF)

Text S1 **List of clinical centres and associated clinicians participating in the OPTIPRIM clinical trial.**
(PDF)
