# Supplementary figures and images for: CD8 T-Cells from Most HIV-Infected Patients Lack Ex Vivo HIV-Suppressive Capacity during Acute and Early Infection
Source: PLoS One. 2013 Mar 29;8(3):e59767. doi: 10.1371/journal.pone.0059767 (PMC3612088; doi:10.1371/journal.pone.0059767)

Figure S1

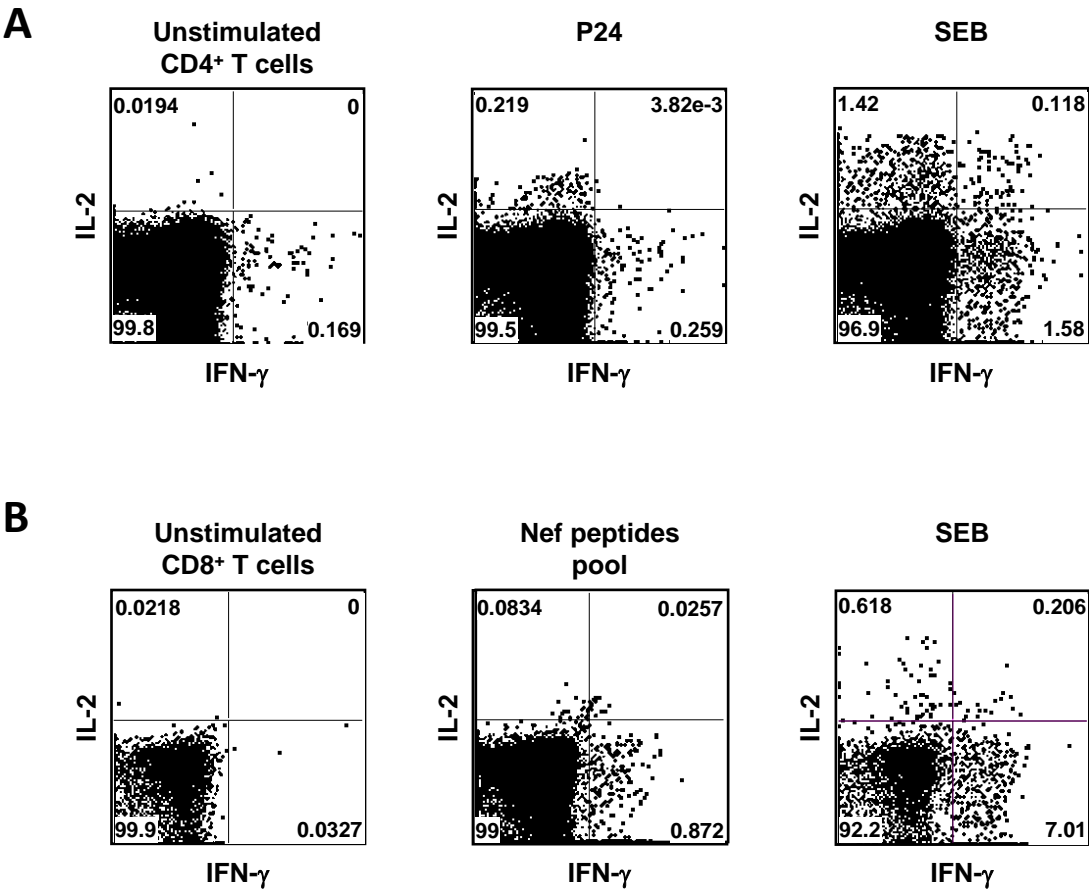

Supplement: Figure S1 — Representative example of ICS experiments showing production of IL-2 and IFNγ by CD4+ T cells (A) or CD8+ T cells (B) from a patient in PHI, in the absence of stimulation or in response to HIV antigens or to SEB superantigen. (PDF) [file pone.0059767.s001.pdf]
