# Supplementary material for: CD8 T-Cells from Most HIV-Infected Patients Lack Ex Vivo HIV-Suppressive Capacity during Acute and Early Infection
Source: PLoS One. 2013 Mar 29;8(3):e59767. doi: 10.1371/journal.pone.0059767 (PMC3612088; doi:10.1371/journal.pone.0059767)
Supplement: Text S1 — List of clinical centres and associated clinicians participating in the OPTIPRIM clinical trial. (PDF) [file pone.0059767.s002.pdf]

OPTIPRIM clinical trial

Brigitte AUTRAN
Ingrid BENARD
Antoine CHERET
Sandrine COUFFIN-CADIERGUES
Cécile GOUJARD
Philippe HALFON
Bruno HOEN
Alain LAFEUILLADE
Caroline LASCOUX-COMBE
Annie LEPALEC
Yann MAZENS
Laurence MEYER
Georges NEMBOT
Daniel OLIVE
Gianfranco PANCINO
Isabelle RAVAUX
Christine ROUZIOUX
Asier SAEZ-CIRION
Juliette SAILLARD
Bruno SPIRE
Catherine TAMALET
Jean-Marc TRELUYER
Alain VENET

CLINICAL CENTERS INCLUDING PATIENTS

Center	Hospital	Place	PI
25	**CHRU Saint Jacques**	**Besançon**	Bruno HOEN
30	**Hôpital St Louis**	**Paris**	Caroline LASCOUX-COMBE
34	**Hôpital Bicêtre**	**Kremlin-Bicêtre**	Cécile GOUJARD
36	**Hôpital Lariboisière**	**Paris**	Agathe RAMI
39	**CHR Aix en Provence**	**Aix en Provence**	Thierry ALLEGRE
48	**Centre médical de l'Institut Pasteur**	**Paris**	Claudine DUVIVIER
49	**Hôpital Tenon**	**Paris**	Laurence SLAMA
56	**Hôpital cochin**	**Paris**	Dominique SALMON
59	**Hôpital Henri Mondor**	**Crétiel**	Yves LEVY
60	**Hôpital Pitié salpétriere**	**Paris**	Christine KATLAMA
61	**Hôpital Pitié salpétriere I**	**Paris**	Anne SIMON
63	**Hôpital St- Louis**	**Paris**	Jean-Michel MOLINA
64	**Hôpital Pierre Zobda Quitman**	**Fort de France Martinique**	Sylvie ABEL
65	**CHU Angers**	**Angers**	Jean-Marie CHENNEBAULT
67	**Hôpital saint André**	**Bordeaux**	Philippe MORLAT
68	**Hôpital Pellegrin**	**Bordeaux**	Jean-Marie RAGNAUD
70	**Hôpital Edouard Herriot**	**Lyon**	Jean-Michel LIVROZET
71/ 98	**Hôpital de la Croix Rousse**	**Lyon**	Patrick MIAILHES
73	**Hôpital Bichat Claude Bernard**	**Paris**	Patrick YENI
75	**Hôpital Gui de Chauliac**	**Montpellier**	Jacques REYNES
76	**Hôpital de l'hôtel Dieu**	**Nantes**	François RAFFI
78	**Hôpital de l'Archet 1**	**Nice**	Anne LEPLATOIS
81	**Hôpital Pontchaillou**	**Rennes**	Faouzi SOUALA
84	**CHU Bretonneau**	**Tours**	Guillaume GRAS
85	**Hôpital Purpan**	**Toulouse**	Lise CUZIN
90	**Centre hospitalier de Tourcoing**	**Tourcoing**	Antoine CHERET
91	**Hôpital de la Conception**	**Marseille**	Isabelle RAVAUX
92	**Hôpital de Bradois de Nancy**	**Nancy**	Thierry MAY
93	**CHU Charles Nicolle**	**Rouen**	Yasmine DEBAB
113	**Hôpital Mignot**	**Le Chesnay**	Alix GREDER BELAN
151	**Hôpital La Grave**	**Toulouse**	François PREVOTEAU DU CLARY
196	**Hôpital Ambroise Paré**	**Marseille**	Patrick PHILIBERT
